# Predictors of Balloon-Assisted Arteriovenous Fistula Maturation: Importance of Intervention Timing and Blood Flow

**DOI:** 10.1159/kdd/adkag002

**Published:** 2026-08-26

**Authors:** Ziming Wan, Hongwei Yang, Qiquan Lai, Xuejing Gao, Ling Chen, Xi Zhang, Hua Gan

**Affiliations:** Department of Nephrology, The First Affiliated Hospital of Chongqing Medical University, Chongqing, China

**Keywords:** Arteriovenous fistula, Balloon-assisted maturation, Influencing factor, Maturation failure

## Abstract

**Introduction:**

Balloon-assisted maturation (BAM) improves the success rate of arteriovenous fistula (AVF) creation. However, the optimal timing of BAM and the factors influencing its success remain unclear. This study aimed to evaluate the clinical success rate of BAM and identify related influencing factors.

**Methods:**

A total of 991 patients undergoing BAM due to AVF immaturity were included (2017–2023) in this study. Data on patient demographics, AVF characteristics, procedural details, brachial artery ultrasound parameters, and preoperative serological indicators were collected. Logistic regression analyses identified factors associated with functional maturation.

**Results:**

The overall assisted maturation rate was 94.15%. An AVF creation-to-BAM interval of <90 days (odds ratio [OR] = 0.413, *p* = 0.006), prolonged thrombin time (OR = 0.209, *p* = 0.007), and elevated D-dimer levels (OR = 0.227, *p* = 0.006) were associated with a reduced maturation rate. A preoperative brachial blood flow ≥300 mL/min (OR = 2.886, *p* = 0.003) and a postoperative flow ≥830 mL/min (OR = 2.590, *p* = 0.002) were protective. Combining all 5 factors yielded an area under the curve of 0.786. In patients aged <65 years, upper arm AVF location (OR = 0.172, *p* = 0.022) and elevated gamma-glutamyl transferase (OR = 0.233, *p* = 0.009) were adverse factors. In patients aged ≥65 years, a low serum calcium level (OR = 0.403, *p* = 0.026) was an additional risk factor. Meanwhile, the AVF creation-to-BAM interval (age <65 years: OR = 0.307, *p* = 0.028; age ≥65 years, OR = 0.423, *p* = 0.045) and postoperative brachial flow (age <65 years: OR = 5.876, *p* = 0.001; age ≥65 years, OR = 2.516, *p* = 0.039) remained independent factors affecting AVF maturation.

**Conclusion:**

BAM is an effective intervention for immature AVFs. A longer interval from AVF creation to BAM and higher postoperative brachial flow are associated with increased AVF maturation rates, especially in younger patients.

## Plain Language Summary

This study presents a comprehensive retrospective analysis of 991 patients who underwent balloon-assisted maturation (BAM) for immature arteriovenous fistulas (AVFs) between 2017 and 2023. Our findings demonstrate a high clinical success rate of BAM (94.15%) and identify key factors influencing AVF maturation, including the timing of BAM intervention, pre- and postoperative brachial artery blood flow, and specific biochemical markers such as thrombin time and D-dimer levels. Notably, we found that an intervention interval of ≥90 days and higher postoperative brachial flow were significantly associated with improved maturation outcomes, with stronger effects observed in younger patients (<65 years). These results challenge the prevailing advocacy for early BAM intervention and emphasize the importance of individualized timing and hemodynamic optimization. Our study offers several novel contributions to the field. First, it provides evidence-based insights into the optimal timing of BAM, a topic still under debate in clinical practice. Second, it highlights the predictive value of postoperative brachial flow over vessel diameter in assessing maturation success. Third, it explores age-stratified factors, revealing distinct risk profiles for younger and older patients, including the novel association between hypocalcemia and reduced maturation in the elderly.

## Introduction

Hemodialysis is the primary treatment for patients with end-stage chronic kidney disease (CKD). Autogenous arteriovenous fistula (AVF) is currently the preferred vascular access for hemodialysis patients because of its advantages, including low infection and hospitalization rates, low mortality, and high patency [1, 2]. However, poor AVF maturation remains a major clinical challenge, with unaided maturation rates ranging from approximately 50%–60% [3, 4].

Balloon-assisted maturation (BAM) is a technique used to promote AVF maturation through one or more postoperative balloon angioplasty procedures. The introduction of BAM has improved the success of AVF creation, particularly for vessels previously considered unsuitable due to small diameters. With angioplasty assistance, clinically functional AVFs can be achieved either after anastomosis or, in select cases, even before anastomosis [5–8]. Moreover, BAM provides durable vascular dilation. Although BAM-matured AVFs exhibit lower blood flow than naturally matured AVFs do 1 year after creation, the relative blood flow ratios between the two groups remain comparable over time [9]. By accelerating maturation, BAM can reduce the dependence on central venous catheters (CVCs) for dialysis, potentially avoiding catheter-related complications [7, 10, 11].

Despite its benefits, the clinical application of BAM is accompanied by ongoing debate and several unresolved issues. First, there is no consensus on the optimal timing for BAMs. Second, the factors influencing functional AVF maturation after BAM remain poorly understood. Addressing these questions is essential for guiding clinical decision-making and standardizing BAM protocols.

Therefore, this study aimed to conduct a large-sample retrospective study to evaluate the success rate and predictors of BAMs in treating AVF maturation failure (MF). By analyzing data from 991 patients, we sought to identify key factors associated with functional maturation, including intervention timing, blood flow parameters, and age-specific considerations, thereby providing evidence to optimize BAM use in clinical practice.

## Methods

### Study Population

This study was a retrospective study approved by the Clinical Research Ethics Committee of the First Affiliated Hospital of Chongqing Medical University (Approval No. K2023-386). The study subjects were patients who underwent BAM for newly created AVFs in this hospital from 2017 to 2023. The inclusion criteria were as follows: (1) age ≥18 years; (2) technically successful BAM procedure; (3) clear postoperative AVF maturation outcomes. During the study period, a total of 1,130 newly created AVFs underwent BAM treatment due to MF caused by stenosis or occlusion. After screening, 9 minors, 1 case of technical BAM failure, 7 cases with unknown outcomes due to death, and 122 lost-to-follow-up cases (loss-to-follow-up rate of 10.80%) were excluded, resulting in 991 cases being included in the final analysis. Diabetic nephropathy (367 cases accounting for 37.03%), glomerulonephritis (363 cases accounting for 36.63%), and hypertensive nephropathy (130 cases accounting for 13.12%) were the main causes of renal failure. All types of lesion locations were included in this study, including anastomotic lesions, inflow tract lesions, and outflow tract lesions. Among these patients, 933 patients achieved functional AVF maturation after BAM, accounting for 94.15% of the patients with known outcomes, and they were included in the assisted maturation (AM) group. The other 58 patients did not achieve maturity and were included in the MF group. The study flowchart is shown in Figure 1.

**Fig. 1.**
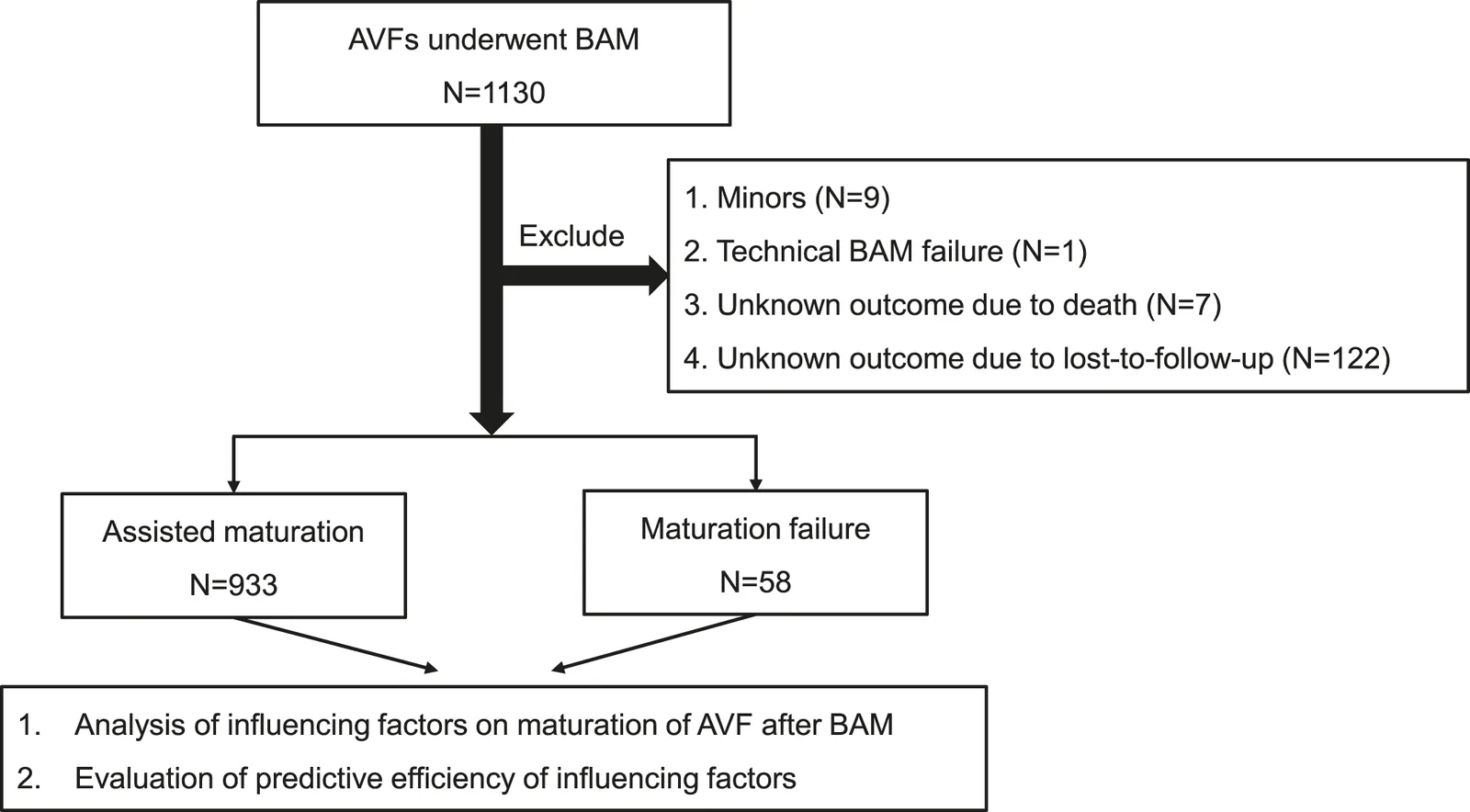
Study flow chart. AVF, arteriovenous fistula; BAM, balloon-assisted maturation.

### Vascular Access Protocol

After physical examination and ultrasound evaluation, patients underwent AVF construction using the end-to-side anastomotic configuration. The maturation status of the fistula was assessed 4–8 weeks post-surgery. According to the Chinese expert guidelines, maturation is defined as meeting the criteria of flow volume ≥500 mL/min, vein diameter ≥5 mm, and vein depth ≤6 mm from the skin surface [1]. MF is defined as the absence of a suitable segment of AVF for cannulation and/or insufficient blood flow (a pump-controlled blood flow <200 mL/min during dialysis) after an adequate waiting period (typically 4–8 weeks after creation) [1]. If issues such as poor fistula dilation, insufficient blood flow, or thrombosis occurred, BAM intervention was considered. A second BAM was indicated if the AVF remained immature at 2 weeks after the initial BAM, and no contraindications were present. Clinical success of BAM is defined if a fistula achieved maturity after one or two BAM procedures. If a fistula could not achieve maturity at 2 weeks after the second BAM attempt, AM was considered a clinical failure, and the patients would choose new vascular access.

### Data Collection and Outcome Definitions

The following data were collected: (1) general demographic information; (2) common comorbidities; (3) fistula location (based on the anatomical site) and type (based on the vessels used); (4) BAM-related information; (5) characteristics of the brachial artery before and after BAM; (6) preoperative serum indicators (online suppl. Table 1; for all online suppl. material, see https://doi.org/10.1159/10.1159/kdd/adkag002). Variables with >20% missing values were excluded from further statistical analysis.

The research outcome was the clinical success of the BAM, which means that the AVF achieved functional maturity after BAM intervention. If the AVF achieves sufficient dialysis blood flow ≥200 mL/min and completes 2 consecutive adequate dialysis sessions post-BAM, it is considered functionally mature [1].

### Statistical Analysis

The data were analyzed using SPSS 22.0, R 4.5.3, and GraphPad Prism 8 software. Classification data are reported as counts and percentages. Denominators for certain variables may differ from the total sample size due to missing data and were noted in the corresponding tables. Quantitative data are reported as medians with interquartile ranges (IQRs). The differences between two groups were compared via the *χ*^*2*^ test, Fisher’s exact probability test, or the Mann-Whitney U test, as appropriate. A two-sided *p* < 0.05 was considered statistically significant. The associations between the research factors and the maturation rate after BAM were sequentially analyzed via univariate logistic regression analysis and multivariate forward (conditional) stepwise regression analysis. For variable selection in multivariate analysis, a threshold of *p* < 0.1 was used in univariate analysis. Because the proportions of missing values were <5% for the variables included in multivariate analysis, complete case analysis was adopted. LASSO (least absolute shrinkage and selection operator) regression with 10-fold cross-validation was conducted as a sensitivity analysis to validate the robustness of predictors under penalized estimation. The LASSO model selected the penalty parameter λ that minimized cross-validated deviance. The results of logistic regression are expressed as odds ratios (ORs) and 95% confidence intervals (CIs). In stratified analysis, the consistency of different strata was assessed via the Breslow-Day test, and the adjusted association was assessed via the Cochran-Mantel-Haenszel test. The predictive value of the research factors for the maturation rate after BAM was evaluated by receiver operating characteristic (ROC) curves and the area under the curve (AUC). Sensitivity (Se) is the proportion of correctly judged mature patients, and specificity (Sp) is the proportion of correctly judged immature patients. The quantitative value corresponding to the maximum Youden index, which is calculated as Se + Sp − 1, is taken as the optimal cutoff value.

## Results

### Patient Characteristics

The baseline characteristics were compared between the AM group and MF group. There was no significant difference in the distribution of sex, body mass index (BMI), diabetes, coronary heart disease, or smoking status between the two groups (*p* > 0.05; Table 1). The most common location of AVF was the wrist, and the most common type was the radiocephalic AVF. No significant difference was noted in the percentages of forearm AVF and RC AVF between the two groups (*p* > 0.05; Table 1). There was no significant difference in median age between the AM group and MF group (61 [IQR: 53, 70] vs. 68 [IQR: 52.5, 74], *p* = 0.094). However, if the patients were divided into two groups on the basis of a cutoff age of 65 years, the proportion of patients aged ≥65 years was significantly higher in the MF group than in the AM group (*p* = 0.004; Table 1; online suppl. Fig. 1).

**Table 1.** Comparison of baseline characteristics

​	Assisted maturation, *N* = 933	MF, *N* = 58	*p*
Male, *N* (%)	462 (49.5)	27 (46.6)	0.661
Age ≥ 65 years, *N* (%)	382 (41.2)	35 (60.3)	0.004
BMI, kg/m^2^	22.2 (19.9, 24.5)	21.8 (19.5, 24.3)	0.328
Diabetes, *N* (%)	430 (46.1)	28 (48.3)	0.746
CHD, *N* (%)	110 (11.8)	8 (13.8)	0.648
Smoking, *N* (%)	256 (27.4)	14 (24.1)	0.584
Forearm AVF, *N* (%)	893 (95.7)	53 (91.4)	0.179^a^
Radiocephalic AVF^b^, *N* (%)	893 (95.7)	54 (93.1)	0.319^a^
Operation time, min	30 (20, 40)	35.5 (26.4, 50.3)	0.005
Cause of primary immaturity, *N* (%)		​	0.007
Stenosis	683 (73.2)	33 (56.9)	​
Occlusion	250 (26.8)	25 (43.1)	​
Creation-BAM interval, days	98 (61, 148)	76 (44, 104)	0.002
Creation-BAM interval group, N (%)		​	0.001
<90 days	420 (45.0)	39 (67.2)	​
≥90 days	513 (55.0)	19 (32.8)	​
Complications^c^, *N* (%)	362 (38.8)	18 (31.0)	0.238
Preoperative diameter, mm	5.10 (4.60, 5.60)	4.85 (4.10, 5.40)	0.021
Preoperative flow, mL/min	303.0 (160.5, 490.0)	194.5 (103.8, 298.0)	<0.001
Postoperative diameter, mm	5.70 (5.20, 6.25)	5.60 (4.78, 6.10)	0.051
Postoperative flow, mL/min	963.0 (808.5, 1160.0)	740.0 (596.5, 1007.8)	<0.001
Preoperative diameter group, *N* (%)		​	0.008
<4.45 mm	199 (21.3)	21 (36.2)	​
≥4.45 mm	734 (78.7)	37 (63.8)	​
Preoperative flow group^d^, *N* (%)		​	<0.001
<300 mL/min	440 (49.5)	41 (75.9)	​
≥300 mL/min	449 (50.5)	13 (24.1)	​
Postoperative diameter group, *N* (%)		​	<0.001
<4.85 mm	140 (15.0)	18 (31.0)	​
≥4.85 mm	793 (85.0)	40 (69.0)	​
Postoperative flow group^d^, *N* (%)		​	<0.001
<830 mL/min	253 (28.2)	31 (59.6)	​
≥830 mL/min	643 (71.8)	21 (40.4)	​
TT group, *N* (%)	​	​	0.006^a^
Normal/low^b^ (≤21 s)	917 (98.3)	53 (91.4)	​
High (>21 s)	16 (1.7)	5 (8.6)	​
D-D group^d^, *N* (%)	​	​	<0.001
Normal (≤0.55 mg/L)	281 (31.2)	5 (8.6)	​
High (>0.55 mg/L)	620 (68.8)	53 (91.4)	​
FDP group^d^, *N* (%)	​	​	0.002
Normal (≤5.0 μg/mL)	729 (80.9)	37 (63.8)	​
High (>5.0 μg/mL)	172 (19.1)	21 (36.2)	​
RBC group^d^, *N* (%)	​	​	0.047
Normal/high^b^ (≥3.8 × 10^12^/L)	400 (44.3)	18 (31.9)	​
Low (<3.8 × 10^12^/L)	502 (55.7)	40 (69.0)	​
MONO group^d^, *N* (%)	​	​	0.039
Normal/low^b^ (≤0.60 × 10^9^/L)	696 (77.3)	38 (65.5)	​
High (>0.60 × 10^9^/L)	204 (22.7)	20 (34.5)	​
GGT group^d^, *N* (%)	​	​	0.043
Normal/low^b^ (≤58 U/L)	780 (85.6)	44 (75.9)	​
High (>58 U/L)	131 (14.4)	14 (24.1)	​

^a^ Fisher exact probability.

^b^ A radiocephalic AVF created at the elbow would be classified as upper arm AVF.

^c^ Angioplasty-related complications include vascular rupture, acute thrombosis, hematomas at the puncture site, guidewire breakage, balloon rupture, and vascular dissection.

^d^ Denominators for these variables vary due to missing data. Missing values were excluded from the denominators for percentage calculations.

MF, maturation failure; BMI, body mass index; CHD, coronary heart disease; AVF, arteriovenous fistula; BAM, balloon-assisted maturation; TT, thrombin time; D-D, D-dimer; FDP, fibrinogen degradation products; RBC, red blood cell; MONO, monocyte; GGT, gamma-glutamyl transferase.

The comparison of BAM-related information is shown in Table 1. The proportion of stenosis-caused primary immaturity in the AM group was higher than that in the MF group (*p* = 0.007). Meanwhile, the operation time in the AM group was also shorter than that in the MF group (*p* = 0.005). Interestingly, the median interval from AVF creation to first BAM in the AM group was 98 (IQR: 61, 148) days, which was significantly longer than the 76 (IQR: 44,104) days in the MF group (*p* = 0.002). An optimal cutoff value of 90 days was selected on the basis of the ROC analysis (online suppl. Fig. 2). Grouping by intervention interval showed that 55.0% of patients in the AM group had an interval of ≥90 days, whereas 67.2% of patients in the MF group had an interval of <90 days (*p* = 0.001).

A total of 362 patients in the AM group experienced angioplasty-related complications (77 arterial ruptures, 214 venous ruptures, 23 acute thrombi, 58 hematomas at the puncture site, 4 guidewire breakages, 4 balloon ruptures, and 1 vascular dissection). Eighteen patients in the MF group experienced complications (4 arterial ruptures, 12 venous ruptures, 5 acute thrombi, and 5 hematomas at the puncture site). All patients with vascular rupture were successfully hemostatic through compression. Small thrombi were not treated specifically, whereas larger thrombi were removed through catheter-based or surgical thrombectomy. Small hematomas were primarily absorbed spontaneously, whereas more severe hematomas were treated with balloon compression for hemostasis, and punctuation was conducted at a different site. In the case of vascular dissection, the guidewire was retracted into the true lumen, repositioned, and repassed. Pseudoaneurysms were relieved by compression. Broken guidewires and ruptured balloons were successfully retracted. No serious consequences occurred. The complication rate did not differ between the two groups (*p* = 0.238; Table 1).

The brachial characteristics before and after BAM were also compared. As shown in Table 1, the AM group had a greater brachial diameter and flow than did the MF group both before and after BAM (*p* < 0.05). The patients were then grouped according to the optimal cutoff values of those characteristics (online suppl. Fig. 3). Compared with the MF group, the AM group had significantly greater proportions of patients with a preoperative brachial diameter ≥4.45 mm, preoperative brachial flow ≥300 mL/min, postoperative brachial diameter ≥4.85 mm, and postoperative brachial flow ≥830 mL/min (*p* < 0.05; Table 1).

Serum indicators before BAM were also analyzed, including renal function, coagulation, routine blood tests, liver function, blood lipids, and calcium and phosphate metabolism. Compared with the AM group, the MF group had higher proportions of high values for 6 indicators, including 3 coagulation indicators (thrombin time [TT], D-dimer [D-D], and fibrinogen degradation products), 2 routine blood indicators (red blood cell and monocyte), and 1 liver function indicator (gamma-glutamyl transferase) (Table 1). No differences were noted in the other indicators (online suppl. Tables 2–7).

### Logistic Regression for Predictive Factors of Fistula Maturation after BAM

Univariate logistic regression analysis was then performed to screen for the factors influencing fistula maturation after BAM. The results are shown in online supplementary Figure 4. A total of 13 factors with *p* values <0.05 were screened out (Table 2) and included in the multivariate analysis. Collinearity among the brachial characteristics was excluded prior to multivariate analysis (online suppl. Table 8). To avoid missing potentially meaningful factors, 3 factors with *p* values between 0.05 and 0.10 were also included in the multivariate analysis. The results of stepwise regression are shown in Table 2. The intervals from AVF creation to BAM <90 days (OR = 0.413, 95% CI: 0.220–0.776, *p* = 0.006), high TT levels (OR = 0.209, 95% CI: 0.067–0.652, *p* = 0.007), and high D-D levels (OR = 0.227, 95% CI: 0.079–0.653, *p* = 0.006) were associated with a decreased maturation rate, whereas larger brachial flow before BAM (OR = 2.886, 95% CI: 1.446–5.760, *p* = 0.003) and after BAM (OR = 2.590, 95% CI: 1.413–4.750, *p* = 0.002) were associated with an increased maturation rate. All 5 predictors from the stepwise model had nonzero coefficients in the LASSO regression model with consistent directional effects.

**Table 2.** Multivariate logistic regression analysis for factors influencing fistula maturation after angioplasty in all patients

Variable	*p*	OR	95% CI	LASSO coefficient
Creation-BAM interval <90 days	0.006	0.413	0.220–0.776	−0.691
Preoperative brachial flow ≥300 mL/min	0.003	2.886	1.446–5.760	0.678
Postoperative brachial flow ≥830 mL/min	0.002	2.590	1.413–4.750	0.713
High TT (>21 s)	0.007	0.209	0.067–0.652	−1.610
High D-D (>0.55 mg/L)	0.006	0.227	0.079–0.653	−0.821

OR, odds ratio; CI, confidence interval; LASSO, least absolute shrinkage and selection operator; BAM, balloon-assisted maturation; TT, thrombin time; D-D, D-dimer.

The performance of these 5 factors in predicting the maturation rate after BAM is shown in Figure 2 and Table 3. For each single factor, postoperative brachial flow had the largest AUC, but the general predictive value of all factors was low, with the AUC not exceeding 0.7. Although high TT levels remained in the final model of logistic regression analysis, ROC analysis revealed that they did not have significant predictive value (*p* = 0.377). Interestingly, TT also had a high sensitivity compared with the other factors, which further led to a higher negative predictive value than that of other factors. Combining all 5 factors yielded an AUC of 0.786 (*p* < 0.001). The model demonstrated excellent calibration, with a mean absolute error of 0.019 and 90% of prediction errors within 3.5% (online suppl. Fig. 5a). Given the class imbalance (94.2% maturation vs. 5.8% failure), model performance was primarily evaluated using precision-recall analysis. The model of 5 combined factors achieved a precision-recall AUC of 0.982, with a recall of 0.995 at the optimal threshold (online suppl. Fig. 5b). These findings indicate that the model of combined factors has excellent discriminative ability for distinguishing MF, while maintaining acceptable overall performance.

**Fig. 2.**
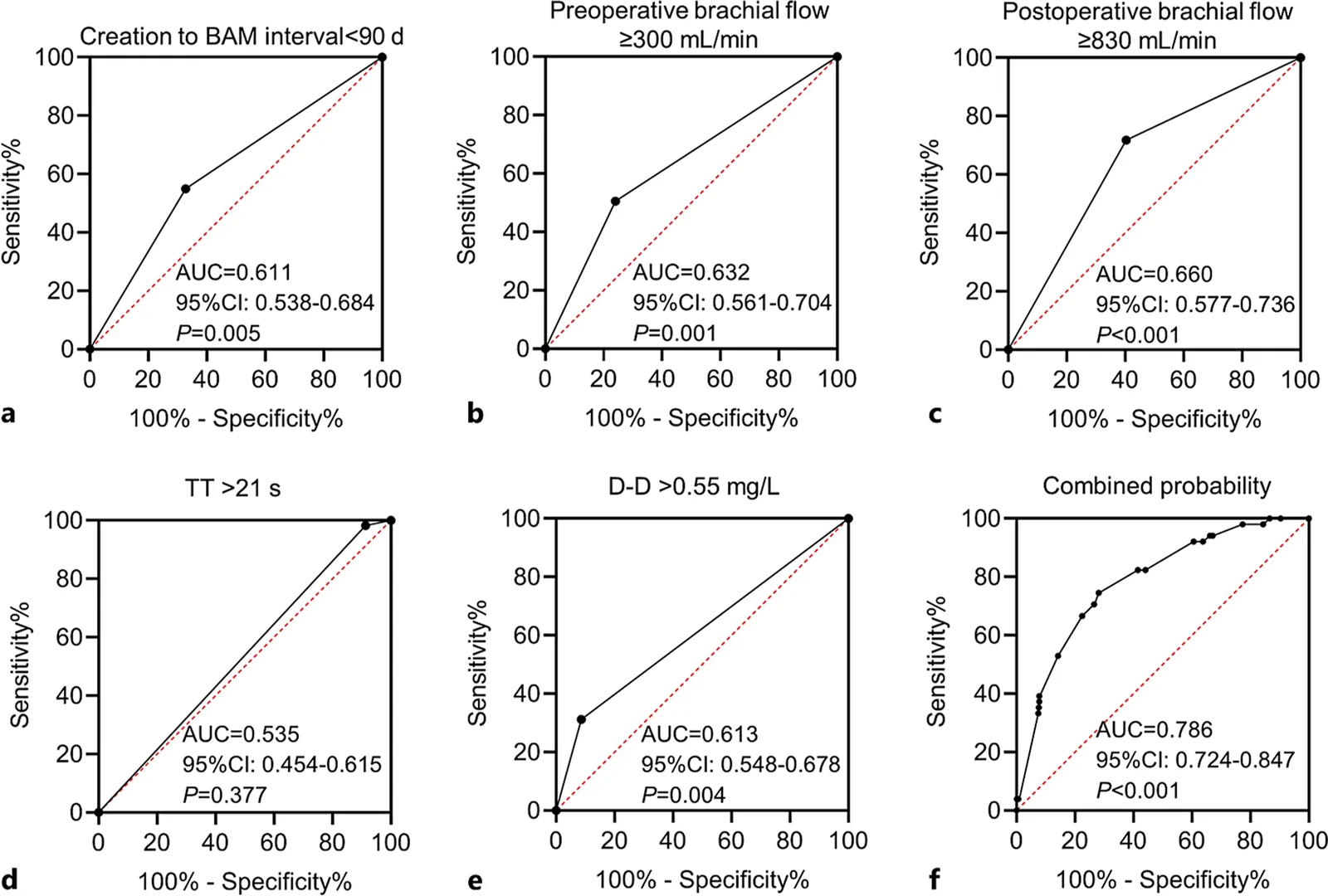
Receiver operating characteristic (ROC) curves of predicting the maturation rate after balloon-assisted maturation (BAM) intervention using fistula creation to BAM interval (**a**), preoperative brachial flow (**b**), postoperative brachial flow (**c**), high thrombin time (TT) value (**d**), high D-dimer (D-D) value (**e**), and all 5 factors combined (**f**). AUC, area under the ROC curve.

**Table 3.** Performance index of various factors for predicting the maturation rate after BAM

​	Se	Sp	PPV	NPV
Creation-BAM interval <90 days	0.550	0.672	0.915	0.036
Preoperative brachial flow ≥300 mL/min	0.505	0.759	0.952	0.095
Postoperative brachial flow ≥830 mL/min	0.718	0.596	0.968	0.109
High TT (>21 s)	0.983	0.086	0.945	0.273
High D-D (>0.55 mg/L)	0.312	0.914	0.983	0.079
All 5 predictors (threshold 0.942)	0.718	0.745	0.979	0.138

BAM, balloon-assisted maturation; Se, sensitivity; Sp, specificity; PPV, positive predictive value; NPV, negative predictive value; TT, thrombin time; D-D, D-dimer.

As shown in Tables 1 and 2, the AVF maturation rate was 91.5% in patients who underwent BAM within 90 days of creation, which was significantly lower than the 96.4% in those who underwent BAM after 90 days (OR = 0.413, *p* = 0.006). The patients were then divided into 4 groups on the basis of the quartiles of the interval from creation to BAM, and the maturation rates after BAM were compared. The maturation rate significantly increased with the prolongation of the intervention interval (trend *χ*^*2*^ = 11.90, *p* = 0.001; Fig. 3). When the continuous intervention interval was analyzed as a covariate, a positive association between the intervention interval and maturation rate (OR = 1.005, 95% CI: 1.000–1.010, *p* = 0.040) was noted by logistic regression analysis.

**Fig. 3.**
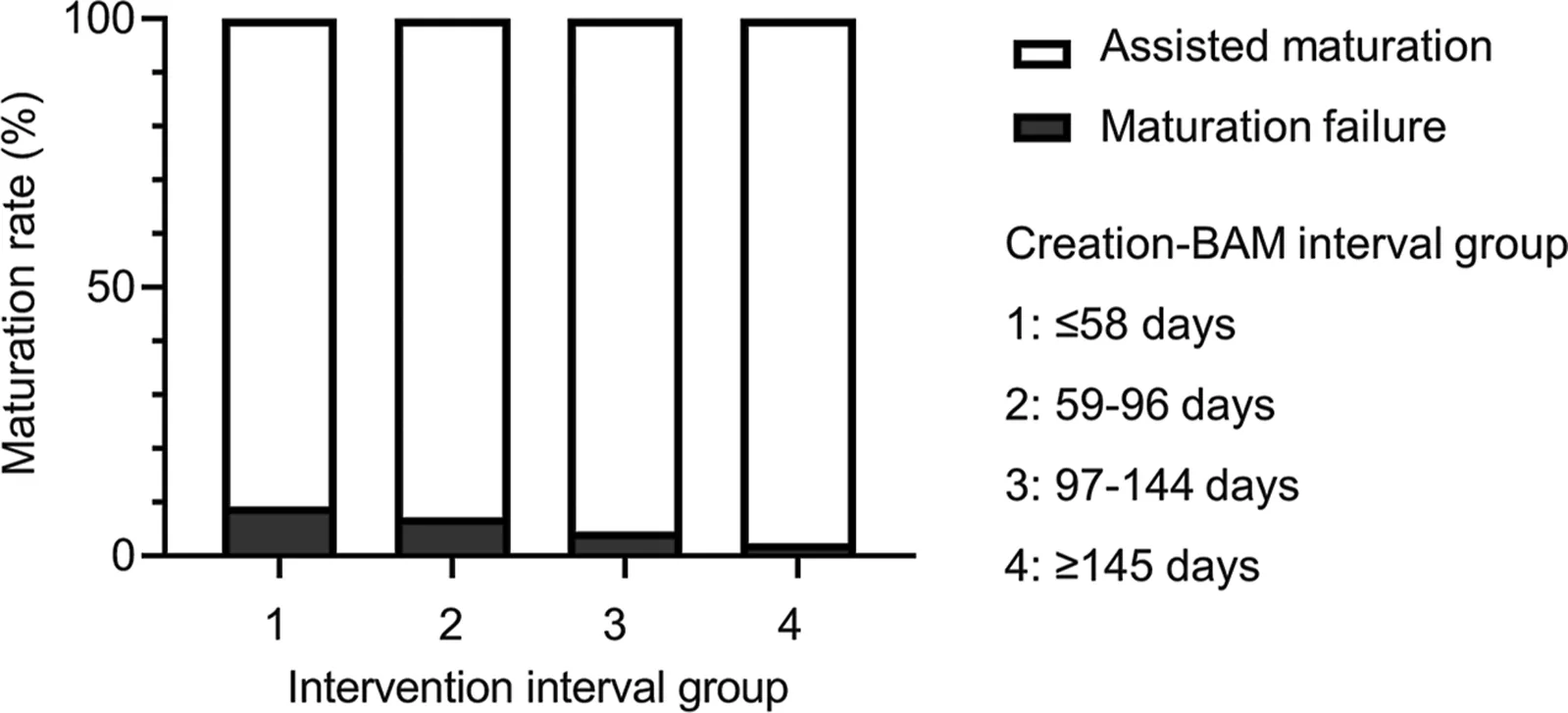
The maturation rate after balloon-assisted maturation (BAM) in groups with different intervention intervals.

### Stratified Analysis of Age

Although age did not remain in the final model in the multivariate logistic analysis, it is a possible confounder affecting other factors. Therefore, age-stratified analysis was also performed to explore whether the influencing factors of maturation after BAM vary across different age subgroups. Of the 991 patients, 574 (57.9%) were aged <65 years with 23 MF patients, and 417 (42.1%) were aged ≥65 years with 35 MF patients. LASSO regression was performed to validate the robustness of predictors selected by stepwise logistic regression.

In the subgroup of patients aged <65 years, multivariate regression analysis was conducted on univariate factors with *p* < 0.01, and the results are presented in Table 4. Upper arm AVF (OR = 0.172, 95% CI: 0.038–0.778, *p* = 0.022), interval from AVF creation to BAM <90 days (OR = 0.307, 95% CI: 0.107–0.882, *p* = 0.028), and high gamma-glutamyl transferase level (OR = 0.233, 95% CI: 0.078–0.694, *p* = 0.009) were associated with decreased maturation rate after BAM. Meanwhile, larger brachial flow after BAM (OR = 5.876, 95% CI: 2.158–15.999, *p* = 0.001) was associated with an increased maturation rate.

**Table 4.** Multivariate logistic regression analysis for factors influencing fistula maturation after angioplasty in different age subgroups

Variable	*p*	OR	95% CI	LASSO coefficient
Age <65 years	​	​	​	​
Upper arm fistula	0.022	0.172	0.038–0.778	−0.124
Creation-BAM interval <90 days	0.028	0.307	0.107–0.882	−0.746
Postoperative brachial flow ≥830 mL/min	0.001	5.876	2.158–15.999	0.859
High GGT (>58 U/L)	0.009	0.233	0.078–0.694	−1.227
Age ≥65 years	​	​	​	​
Creation-BAM interval <90 days	0.045	0.423	0.182–0.982	−0.375
Preoperative brachial flow ≥300 mL/min	0.039	2.516	1.047–6.047	0.576
Postoperative brachial flow ≥830 mL/min	0.034	2.352	1.066–5.186	0.346
High TT (>21 s)	0.019	0.151	0.031–0.736	−1.853
Low calcium (<2.1 mM)	0.026	0.403	0.181–0.897	−0.183

OR, odds ratio; CI, confidence interval; LASSO, least absolute shrinkage and selection operator; BAM, balloon-assisted maturation; TT, thrombin time; GGT, gamma-glutamyl transferase.

In the subgroup of patients aged ≥65 years, the results of multivariate logistic regression revealed different findings (Table 4). An interval from AVF creation to BAM <90 days (OR = 0.423, 95% CI: 0.182–0.982, *p* = 0.045), a high TT level (OR = 0.151, 95% CI: 0.031–0.736, *p* = 0.019), and a low calcium level (OR = 0.403, 95% CI: 0.181–0.897, *p* = 0.026) were associated with a decreased maturation rate after BAM. Greater brachial flow before (OR = 2.516, 95% CI: 1.047–6.047, *p* = 0.039) and after (OR = 2.352, 95% CI: 1.066–5.186, *p* = 0.034) BAM was associated with an increased maturation rate.

Two common influencing factors in both subgroups were the intervention interval and postoperative brachial flow. Their association with maturation after BAM was stronger in the subgroup of patients <65 years. These two factors were also retained in the final model of the multivariate regression before age stratification (Table 2). After adjusting for age, the OR of the intervention interval <90 days was 0.430 (95% CI: 0.229–0.709, *p* = 0.002) and the OR of postoperative brachial flow ≥830 mL/min was 3.507 (95% CI: 1.978–6.217, *p* < 0.001). The detailed results of the age stratification analysis are shown in online supplementary Figures 6–13.

## Discussion

This study explored the factors influencing the AVF maturation rate after BAM. The results revealed that among 991 patients, the overall maturation rate after BAM was 94.15%. Combining the results before and after age stratification, the interval from AVF creation to BAM and brachial artery flow after BAM were identified as the two most important predictors. Later BAM intervention and higher brachial flow after BAM were associated with a higher AVF maturation rate, and these two factors had a stronger association with the maturation rate in individuals aged <65 years.

Currently, there is no universal consensus on the optimal timing for AM interventions. Although guidelines recommend maturity assessment at 4–8 weeks after creation [1], the timing of maturity assessment and the timing for intervention are two distinct issues. Our findings suggest that extending the observation window to 12 weeks before initiating salvage intervention may be beneficial, as a longer creation to BAM interval was associated with a higher maturation rate. This finding appears to contradict the current advocacy for early intervention in poorly maturing AVFs. Some studies suggest that it is appropriate to perform BAM 6 weeks after fistula creation, but these studies did not compare maturation rates under different intervention timings [9, 12]. Proponents of early intervention argue that BAM can shorten the maturation time, thereby reducing the CVC dependence and related complications [13]. However, this benefit presupposes that early intervention can achieve a mature AVF. If early intervention fails, the premise of shortened CVC-dependent duration actually does not hold. Patients may instead undergo revision or new access creation, resulting in prolonged CVC dependence. Moreover, some studies suggest that BAM does not shorten the maturation time [14]. Other researchers advocate early intervention because signs of maturation (such as changes in vessel diameter and blood flow) can be observed 4–6 weeks after AVF creation. If there are signs of poor maturation, intervention is necessary; otherwise, stenosis may progress to occlusion, thus losing the opportunity for intervention [15]. However, this inference seems far-fetched at present. With the application of ultrasound guidance and improvements in medical skills, the clinical success rate of BAM for occluded AVFs can reach 90% (such as in our center). Moreover, the data from this study indicate that there was no difference in intervention timing between occlusive and stenotic conditions (97 [IQR: 51–145] days vs. 96 [IQR: 64–145] days, *p* = 0.141). Thus, the presumed advantages of early intervention are questionable, and the relationship between BAM timing and the postoperative AVF maturation rate requires further investigation.

Another key finding is that brachial diameter and flow before and after BAM are associated with the post-BAM maturation rate, with brachial flow post-BAM having the highest predictive value. Consistent with Hakim et al. [16], who reported that blood flow has the highest diagnostic value among the “three 6” criteria, our results suggest that AVF blood flow (generally measured as brachial flow) is a robust predictor. Another study by Kim et al. [17] did not find an association between pre-BAM fistula flow and the post-BAM maturation rate. Consistently, our study subgroup analysis revealed that pre-BAM brachial flow was not retained in the final regression model for patients aged <65 years but was retained for those aged ≥65 years. We speculate that this difference may be related to decreased vascular elasticity in elderly patients. Although brachial flow before BAM did not differ between the two subgroups (278.5 [IQR: 158.0, 491.3] mL/min vs. 300.5 [IQR: 157.3, 463.3] mL/min, *p* = 0.998), the increase in brachial flow was greater in younger patients (643.0 [IQR: 479.3, 841.8] mL/min vs. 601.0 [IQR: 434.5, 785.5] mL/min, *p* = 0.021). From a clinical perspective, postoperative blood flow better represents the environmental conditions when the AVF matures and is more reasonable for predicting the maturation rate. It is necessary to emphasize that postoperative blood flow should solely be interpreted as a predictor for maturation. Our findings should not be interpreted as supporting the use of more aggressive balloon dilation for higher postoperative flow because they did not establish that deliberately maximizing postoperative blood flow would improve maturation. Moreover, larger balloons (≥8 mm) increase the risk of complications [12, 18]. A balloon size of 4–5 mm is most commonly used in our practice. Furthermore, our data showed that there was only a weak correlation between vessel diameter and blood flow. Therefore, using large-sized balloons in pursuit of high postoperative flow is a misinterpretation of our results. If postoperative flow is below a certain threshold, it may prompt early surveillance rather than immediate reintervention.

Age was significantly associated with the maturation rate in univariate analysis but was not retained in the final multivariate model. This result indicates that advanced age is not a contraindication for BAM intervention. Age stratification analysis revealed complex confounding and interaction effects (with total cholesterol and serum calcium). Two factors, namely, the interval from AVF creation to BAM and the brachial flow after BAM, can be considered for patients of all ages, with a stronger association in younger patients. For older patients, TT levels and serum calcium levels can also be considered. CKD patients are more susceptible to disorders in the metabolism of calcium, and hypocalcemia is associated with accelerated progression of CKD and increases the mortality risk [19–21]. To the best of our knowledge, the association between hypocalcemia and the AVF maturation rate has not been reported. Our results provide new evidence for the importance of maintaining calcium balance in CKD patients. Regarding serum indicators (TT and D-D), we acknowledge that single-time-point measurements are not ideal for predicting long-term outcomes due to fluctuations from diet and medications. This may be one of the reasons why current research on AVF maturity generally does not involve serum indicators. ROC analysis showed that the predictive value of the serum markers was very poor. However, it is worth mentioning that TT had a higher negative predictive value, which may help identify patients at a higher risk of failure and plan ahead for alternative measures (such as switching to grafts or CVCs instead of repeated BAMs).

As a retrospective study, this research has inherent limitations that are difficult to overcome. First, there is information bias due to missing data, especially in the medical records collected in the early stage. With some patients having died or being lost to follow-up, tracing these cases is no longer possible. Indicators with missing values exceeding 20% were excluded from the analysis. Second, there is selection bias caused by loss to follow-up. The loss-to-follow-up rate in this study was 10.8%, which is within an acceptable range, indicating good internal validity. Third, the outcome number of MF was relatively small, especially after age stratification, which may impair the statistical power of logistic regression. Although LASSO regression confirmed robustness, the results should still be interpreted cautiously and be validated in larger independent cohorts. Fourth, the intervention timing could not be assigned deliberately, which would require a clinical trial. The AVFs that were identified as MF at 4–8 weeks may have simply been premature. Some AVFs might have matured spontaneously later without any intervention. Meanwhile, although the proportion of occlusive lesions did not differ between the early and late intervention groups (28.5% vs. 27.1%. *p* = 0.606), the confounding effect by indication cannot be excluded entirely. Moreover, although this study included all types of lesion locations, it was unable to perform a comparative analysis of BAM success rates by lesion location. In patients with occlusive lesions, only approximately 30% had a lesion site clearly identifiable by ultrasound imaging. In our center, over 90% of patients requiring assistance for maturation underwent BAM. Thus, our findings are generalizable to the broader population of patients with AVF MF. While this study focuses on predictors of BAM success, we acknowledge that long-term patency and durability are important. Long-term patency data have been analyzed separately and will be reported in a companion paper due to length limitations and different study designs.

In conclusion, BAM has a good clinical effect on increasing the AVF maturation rate. The interval from AVF creation to BAM and post-BAM brachial flow are the two most significant factors associated with the maturation rate after BAM, and the association is relatively stronger in patients aged <65 years. Clinical decision-making should comprehensively consider intervention timing, hemodynamic parameters, and biochemical indicators to optimize outcomes.

## Statement of Ethics

The study was approved by the Clinical Research Ethics Committee of the First Affiliated Hospital of Chongqing Medical University (Approval No. K2023-386). Written informed consent was waived by the same committee because of the retrospective nature of this study.

## Conflict of Interest Statement

The authors have no conflicts of interest to declare.

## Funding Sources

The research did not receive any external funding.

## Author Contributions

Z.W. and H.G. designed the study, interpreted the results, and wrote the draft. Z.W., H.Y., Q.L., X.G., L.C., and X.Z. collected and checked the data. Z.W. and H.Y. analyzed the data. All authors reviewed and approved the manuscript.

## Data Availability

The data that support the findings of this study are not publicly available due to privacy reasons but are available from the corresponding author upon reasonable request.
